# Effects of cigarette smoking on the oral microbiome in adolescents

**DOI:** 10.1038/s41598-025-32650-2

**Published:** 2026-01-10

**Authors:** Paula Schaefer-Dreyer, Wiebke Behrens, Andreas Winkel, Philipp-Cornelius Pott, Mira Paulsen, Nils Stanislawski, Fatma Tanisik, Anette Melk, Bernhard Magnus Wilhelm Schmidt, Henning Lucas, Stefanie Heiden, Norman Klopp, Thomas Illig, Holger Blume, Cornelia Blume, Ines Yang, Meike Stiesch

**Affiliations:** 1 Department of Prosthetic Dentistry and Biomedical Materials Science, Hannover Medical School, Carl-Neuberg-Str. 1, 30625 Hannover, Germany; 2 Lower Saxony Centre for Biomedical Engineering, Implant Research and Development (NIFE), Hannover, Germany; 3 Department of Pediatric Kidney, Liver, and Metabolic Diseases, Hannover Medical School, Hannover, Germany; 4 Institute of Microelectronic Systems, Leibniz University Hannover, Hannover, Germany; 5 Department of Nephrology and Hypertension, Hannover Medical School, Hannover, Germany; 6 Institute of Innovation Research, Technology Management & Entrepreneurship, Leibniz University Hannover, Hannover, Germany; 7 Hannover Unified Biobank, Hannover Medical School, Hannover, Germany; 8 Institute for Technical Chemistry, Leibniz University Hannover, Hannover, Germany

**Keywords:** Microbiome, Community composition, Bacterial abundance, Microbiota of the buccal mucosa, Adolescents, Full-length 16S rRNA amplicon sequencing, Cigarette smoking, Early-onset effect of smoking, Diseases, Medical research, Microbiology

## Abstract

Smoking, a risk factor for periodontitis and peri-implantitis, is associated with shifts in the oral microbiome (OM) composition. Although smoking habits are almost always established before adulthood, data on effects of smoking on the OM in adolescents is rare. The aim of this study was to investigate the early impact of smoking on the OM composition in pupils. The adolescent cohort, aged 14–20, comprised 98 smokers and 98 non-smokers matched for several physiological co-variates. Buccal swabs were analysed for OM composition using high-throughput sequencing of the full-length 16 S rRNA gene targeting species-level resolution. Parameters of bacterial diversity and abundance of individual bacterial taxa were related to information on smoking. The microbiome dataset contained 733 species-level taxa. *Streptococcus*, *Rothia*, and *Haemophilus* dominated both groups, smokers and non-smokers. Smoking exerted a discernible influence on the overall microbial composition as measured by weighted UniFrac distances. The number of species-level bacterial taxa was significantly higher in individual smokers compared to non-smokers. Furthermore, several taxa, including known pathogens, exhibited significant differences in abundance between the two groups. The genera *Veillonella*, and *Actinomyces*, as well as and multiple *Actinomyces* species, *Dialister invisus*, *Atopobium parvulum*, *Streptococcus mutans* and *Prevotella melaninogenica* were significantly more abundant in smokers. Our findings indicated an early onset of smoking-related changes already in the oral microbiome of adolescents.

**Supplementary Information:**

The online version contains supplementary material available at 10.1038/s41598-025-32650-2.

## Introduction

The human oral microbiome contains over 700 known species of bacteria^1,2^.Metagenomic data also suggest that several thousand additional species remain largely undescribed^3,4^. A single individual oral sample typically contains a fraction of this spectrum, usually between 50 and 200 different bacterial species^5,6^. These bacteria fulfill a variety of functions and many of them are critical for maintaining oral health^7^. A shift in the oral microbiome, known as dysbiosis, is a critical step in the development of oral diseases such as periodontitis and dental caries^8–11^. Smoking is associated with shifts in the composition of the oral microbiome^12–15^ and can contribute to the development and progression of periodontitis^16–18^ and oral peri-implantitis^19–21^. Studies in adults show that smoking changes several conditions for bacterial growth in the oral cavity^22^. These alterations include complex effects on the immune system resulting in suppressed immune cell function^23,24^ but increased pro-inflammatory mediators^25,26^. Additionally, the oxygen tension in gingival pockets, which reflects the partial pressure of oxygen available for microbial metabolism, and saliva pH are reduced in smokers^27–29^. Cigarettes were also shown to directly contain bacteria, including several taxa with pathogenic potential, and might therefore contribute to the accumulation of these taxa in the oral microbiome of smokers^30^. These smoking-related effects seem to favour a pathogenic shift in the oral microbiome which has been observed for adult smokers so far^12,15,22,31^.

While most studies on the effects of smoking are conducted in adult long-term smokers, smoking habits are mostly already established before the age of 18^32^. In 2022, approximately 16% of German 14- to 17-year-old adolescents and 40.8% of 18- to 24-year-old adolescents classified as current smokers^33,34^. While it appears likely that the first smoking-related changes of the oral microbiome will develop with the beginning of smoking the oral microbiome of adolescent smokers is severely understudied. We conducted a comprehensive assessment of oral microbiome community compositions and bacterial abundances in 196 pupils, including 98 smokers and 98 matched non-smokers to improve the understanding of the impact of smoking on the oral microbiome in adolescents.

## Materials and methods

### Study population

This study was carried out in the framework of the longitudinal Transmission Analytic COVID-19 (TRAC-19) study, which analysed infections, behavioural patterns and vaccination hesitancy during the SARS-CoV-2 pandemic in two schools within a German city^35^. Participation in the study was voluntary and required the written consent of the participants and the legal guardians of minors. The study was approved by the Ethics Committee of the Hannover Medical School (Ethical Vote No. 9085_BO_S_2020) and is in accordance with the Declaration of Helsinki^35^.

Samples for this sub-study on the oral microbiome were collected on-site during school hours from June to December 2020 from secondary-school pupils. All included participants were between 14 and 20 years of age, indicated that they brushed their teeth at least twice a day and did not take regular medication except for hormonal contraceptives or either paracetamol and/or ibuprofen and/or lactase on less than one occasion a week. Participants who had taken antibiotics were excluded from the study. All participants reported good oral hygiene practices and the absence of oral health issues.

A total of 98 samples were collected from current smokers. These smoking participants were matched one-to-one with similar participants who reported that they had never smoked, resulting in a combined dataset consisting of 196 participants. The following criteria were taken into account for matching: sex, age, height, BMI, and use of hormonal contraceptives. Successfully balanced matching was verified as detailed in Table 1. Independent t-test, Chi-square and Fisher exact test were calculated with the R package stats (version 4.1.0).

**Table 1:** Demographic and other selected characteristics of the study participants. To obtain two balanced groups, each smoking participant was matched with a non-smoker by age, sex, height, BMI, and hormonal contraceptive use.

	Non-smoke	Smoker	Test	Test result
n	98	98		
Age, years, median (Q1–Q3)	**17.06** (16.22–18.04)	**17.50** (16.52–18.46)	*t* test	ns
Sex, n (%)			Chi-square test	ns
Female	**53** (54.1)	**53** (54.1)		
Male	**45** (45.9)	**45** (45.9)		
Weight, kg, median (Q1–Q3)	**65.00** (60.00–72.00)	**65.00** (60.00–72.75)	*t* test	ns
Height, cm, median (Q1–Q3)	**174.0** (167.0–181.8)	**174.5** (168.0–181.8)	*t* test	ns
BMI, kg/m^2^, median (Q1–Q3)	**21.49** (20.20–22.80)	**21.26** (20.00–23.01)	*t* test	ns
Conventional cigarettes, n (%)	**0** (0)	**81** (82.65)		
Electronic cigarettes, n (%)	**0** (0)	**48** (48.98)		
Hormonal contraceptives^a^	**13**	**13**	Chi-square test	ns
Regular medication intake^b^, n	**1**	**4**	Fisher exact test	ns

Q1: Lower quartile, Q3: upper quartile, BMI: body mass index, a: contraceptive pill or other hormonal contraceptives, b: paracetamol, ibuprofen and/or Lactase, in all cases less than 1 use per week, ns: not significant (*p* > 0.05).

### Data and sample collection

To analyse the oral microbiome, samples were taken from the buccal mucosa of all participants. This was done using sterile and DNA-free cotton swabs (Sarstedt, Nuembrecht, Germany), which were wiped three times for approximately ten seconds on the inside of each cheek. Afterwards, each cotton swab was transferred to 1 ml DNA/RNA shield (Zymo Research, Freiburg, Germany), and stored at − 80 °C. Health data of the participants were collected with a questionnaire (see Supplementary Table 1). The following parameters were queried: age, sex, weight, height, oral health status, tooth brushing frequency, known chronic diseases, and type and frequency of regular medication intake including hormonal contraceptives. In addition, several questions defined the smoking behaviour. Students self-reported whether they had ever smoked before and if so, whether they consumed conventional cigarettes, electronic cigarettes or both. Smoking pupils classified their smoking frequency with one of six categories from “less than once/month” to “more than 20 cigarettes/day”. The majority of smoking pupils consumed conventional cigarettes (81 out of 98), but also e-cigarettes were frequently used (48 out of 98). Bacterial communities of smokers using only conventional, only e-cigarettes, or a combination showed no significant differences (see Supplementary Fig. 3). Therefore, we combined all smokers of conventional and e-cigarettes into the single category “smokers”.

### Oral microbiota analyses

#### Sample preparation and SMRT full-length 16 S rDNA amplicon sequencing

DNA was extracted under DNA-free conditions using a combination of mechanical disruption and column-based DNA isolation (see Supplementary Methods). Bacterial 16 S rDNA genes were amplified using the bacteria-specific primer pair 27 F (AGRGTTYGATYMTGGCTCAG) and 1492R (RGYTACCTTGTTACGACTT), sequenced using PacBio Sequel technology, and analysed with an in-house pipeline (see Supplementary Methods).

#### Bacterial spike-ins

To allow for absolute quantification of bacterial cells of oral microbiota species, and to test for sample-specific variations in DNA extraction efficiency for bacterial species with different cell wall characteristics, 2.8 µl ZymoBIOMICS Spike-in Control I (Zymo Research, Freiburg, Germany) were added to each sample as well as to every empty control. This spike-in control contains specified cell numbers of the bacterial species *Imtechella halotolerans* and *Allobacillus halotolerans*, which are not members of the human microbiome.

#### Negative controls

To control for potential laboratory or chemical contaminants, empty samples consisting of clean cotton swabs in 1 ml DNA/RNA shield were processed in parallel during all steps of sample preparation, from DNA extraction to library preparation and sequencing (see Supplementary Table 2 for a list of potential contaminants).

#### Microbiota statistics

Alpha-diversity metrics (number of observed species-level taxa, Shannon index, evenness) were calculated for comparison of intra-sample diversity. To allow inter-sample comparability for these metrics, all samples were subsampled to 3,040 sequence reads. One sample and its corresponding match were excluded from this analysis (97 pairs remained) because it contained only 1,316 sequences. The observed number of species-level taxa and the Shannon index were determined with the functions tax_glom and estimate_richness of the R package phyloseq (version 1.36.0)^36^. Based on this, evenness was calculated as follows: Shannon index/log_*e*_(number of observed species-level taxa). Descriptive statistics, tests for homogeneity of variance and normal distribution, independent *t*-test, and effect sizes (Cohen’s d) were calculated with the R packages car (version 3.1.1), stats (version 4.1.0) and lsr (version 0.5.2).

The sample-specific extraction efficiency index was calculated based on the spike-in species ratio (see Supplementary Methods, Supplementary Fig. 1). All PERMANOVA and DESeq2 analyses were performed while controlling for this sample-specific technical variable.

Analyses of beta-diversity were based on weighted UniFrac distances^37^. The underlying phylogenetic tree was inferred on the basis of an infernal 1.1.2 alignment^38^ using the double-precision version of FastTree 2^39^. Principal Coordinates Analyses (PCoA) as a multivariate, unconstrained ordination method were calculated using the R package phyloseq (version 1.36.0). Significance of overall group differences were assessed using PERMANOVA as implemented in the adonis2 function of the R package vegan (version 2.6.4). The following possible non-technical co-variates were included in the PERMANOVA calculations as indicated: age in years, BMI, sex, hormonal contraceptives and percentage of Imtechella reads as proxy for the number of bacterial cells in the original sample. Differences in averages of pairwise weighted UniFrac distances between all samples of selected groups were calculated as independent t-test or ANOVA with R package stats (version 4.1.0). Association of individual taxa with the smoking behaviour (smoking/non-smoking) were tested with DESeq2 controlling for sample-specific extraction efficiency index^40^.

## Results

### Characteristics of the study population

A group of 98 smokers (age 14.2–20.3 years) and 98 matched non-smokers (age 14.2–19.4 years) was analysed (Table 1). Pupils in each study group had a median age of 17 years, normal weight (median BMI of 21 kg/m^2^ in both), and used hormonal contraceptives in 13 cases. Although sexes were balanced between smokers and non-smokers, more study participants were female than male. Four participants, all from the group of smokers, reported use of either paracetamol and/or ibuprofen and/or lactase on less than one occasion a week.

Smoking frequencies that were reported by the pupils covered five categories ranging from “less than once/month” (category 1) to “every day” (category 5) (Supplementary Fig. 2). No participant met the criterion for heavy smoking (more than 20 cigarettes/day, category 6).

### Smoking increases the number of species-level taxa

**Fig. 1:**
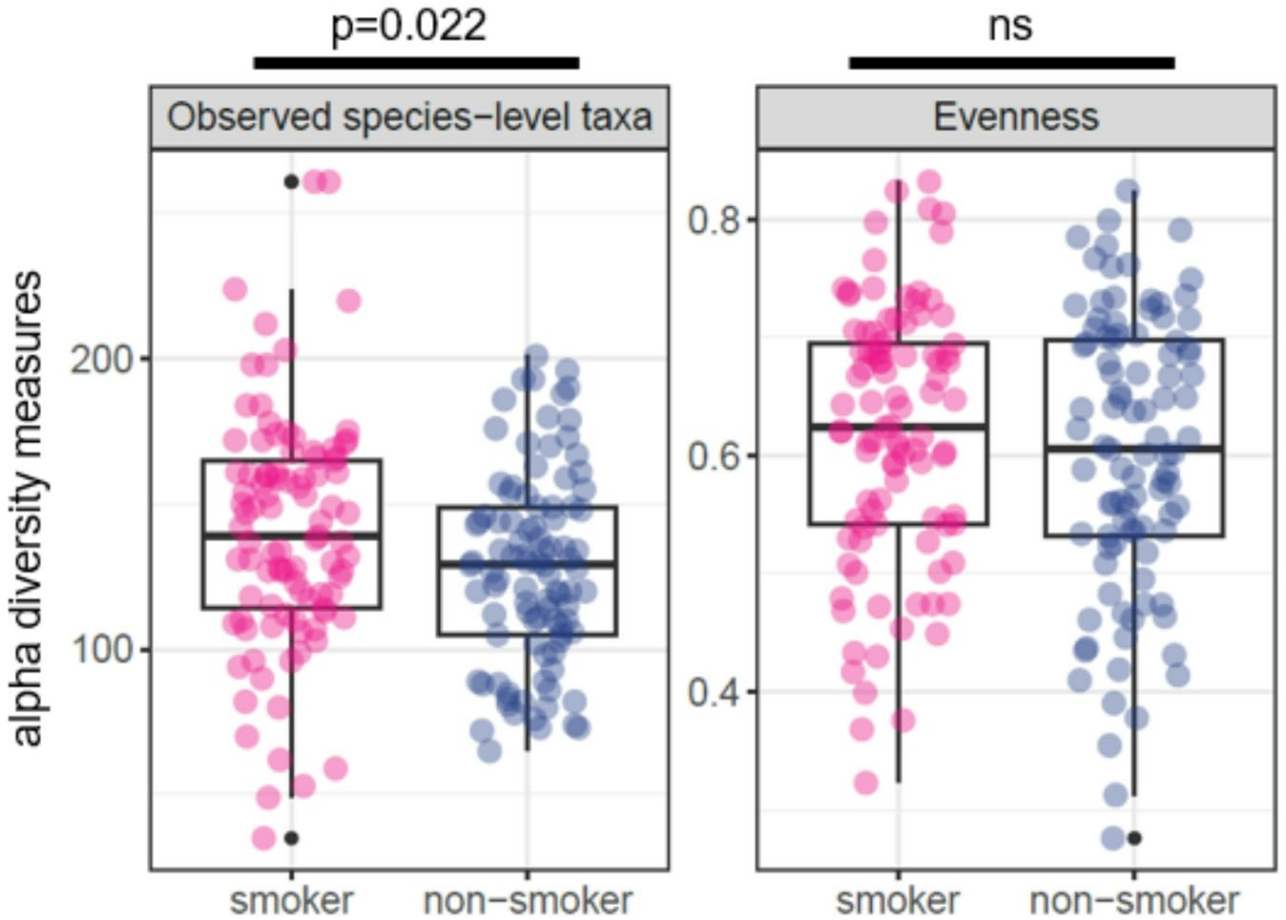
Alpha diversity measurements on species-level in non-smokers and smokers. Number of observed species-level taxa (left panel) and calculated evenness (right panel) are displayed as boxes with median (50th percentile), 25th and 75th percentiles, and whiskers that reach to 1.5 times of the interquartile ranges. Outliers are depicted as black dot.

To compare the diversity of bacterial communities within each sample (alpha-diversity) between smokers and non-smokers, samples were rarefied to equal sequence numbers before calculating richness (number of observed species-level taxa), evenness, and Shannon index. The observed numbers of species-level taxa were significantly higher in smokers registering a mean of 140 ± 41 compared to 127 ± 33 in non-smokers (*t*-test, *p* = 0.022, Fig. 1), although the effect was small (Cohen’s d = 0.33). No significant differences were observed for evenness and Shannon index. The smoking frequency was not associated with significant differences in alpha-diversity (ANOVA, *p* > 0.5). Comparison of total cell numbers of smokers and non-smokers indicated that both groups of buccal samples contained similar amounts of bacterial cells (*t*-test: *p* > 0.05, Supplementary Fig. 4).

### The bacterial composition differs between smokers and non-smokers

**Fig. 2:**
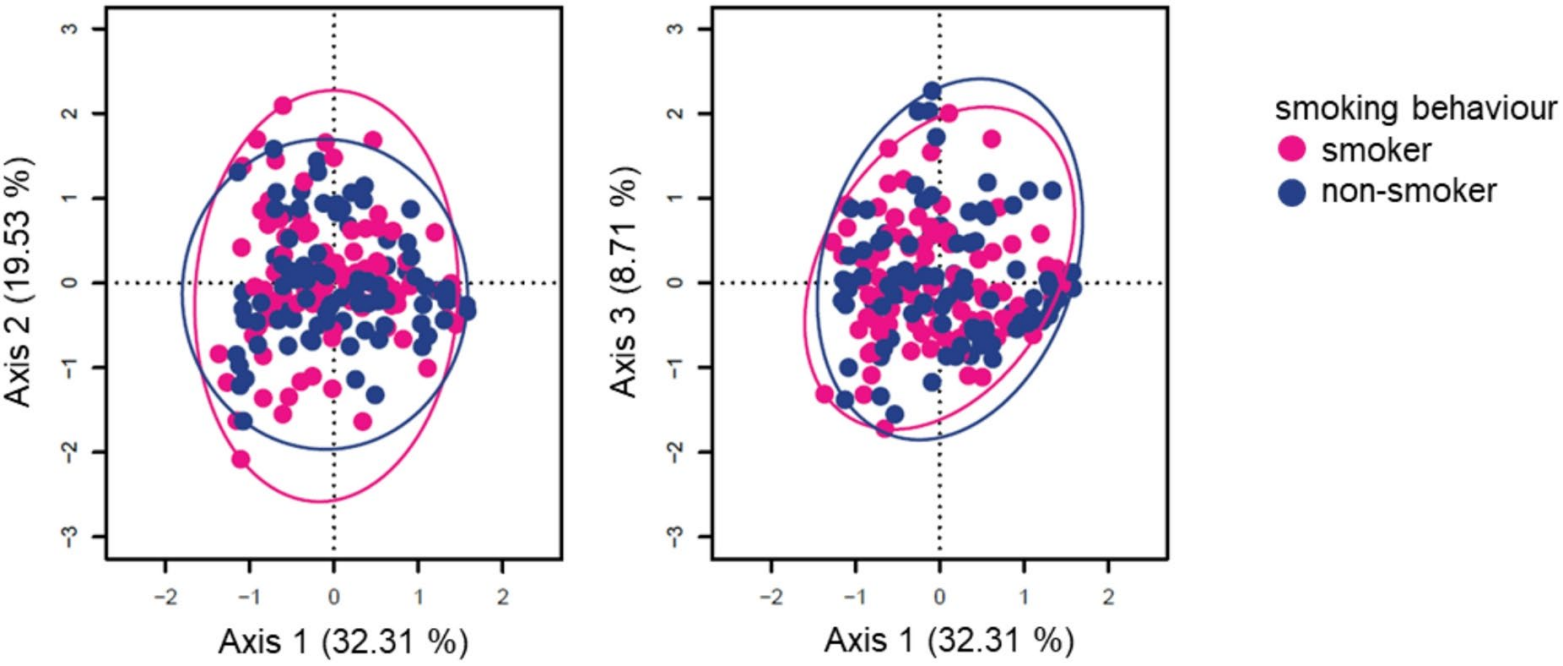
PCoA on weighted UniFrac distances. Samples are coloured according to smoking behaviour (non-smokers: blue, smokers: pink). Ellipses enclose all points in the respective group.

The overall composition of the microbiome differed significantly between smokers and non-smokers (PERMANOVA based on weighted UniFrac distances including only the technical co-variate: *p* = 0.026, R^2^ = 0.013; including technical and non-technical co-variates: *p* = 0.038, R^2^ = 0.011). A principal coordinates analysis indicated a corresponding small shift in smoking behaviour-dependent clustering of samples (Fig. 2). Pairwise weighted UniFrac distances between all samples within the group of smokers did not significantly differ from distances within the group of non-smokers (ANOVA, Supplementary Fig. 5). The microbiome composition did not significantly differ between groups of different smoking frequencies (PERMANOVA based on weighted UniFrac distances, *p* > 0.5) which are characterized by varying, small subgroup sizes (Supplementary Fig. 2).

### Most abundant taxa in the Microbiome of adolescents

**Fig. 3:**
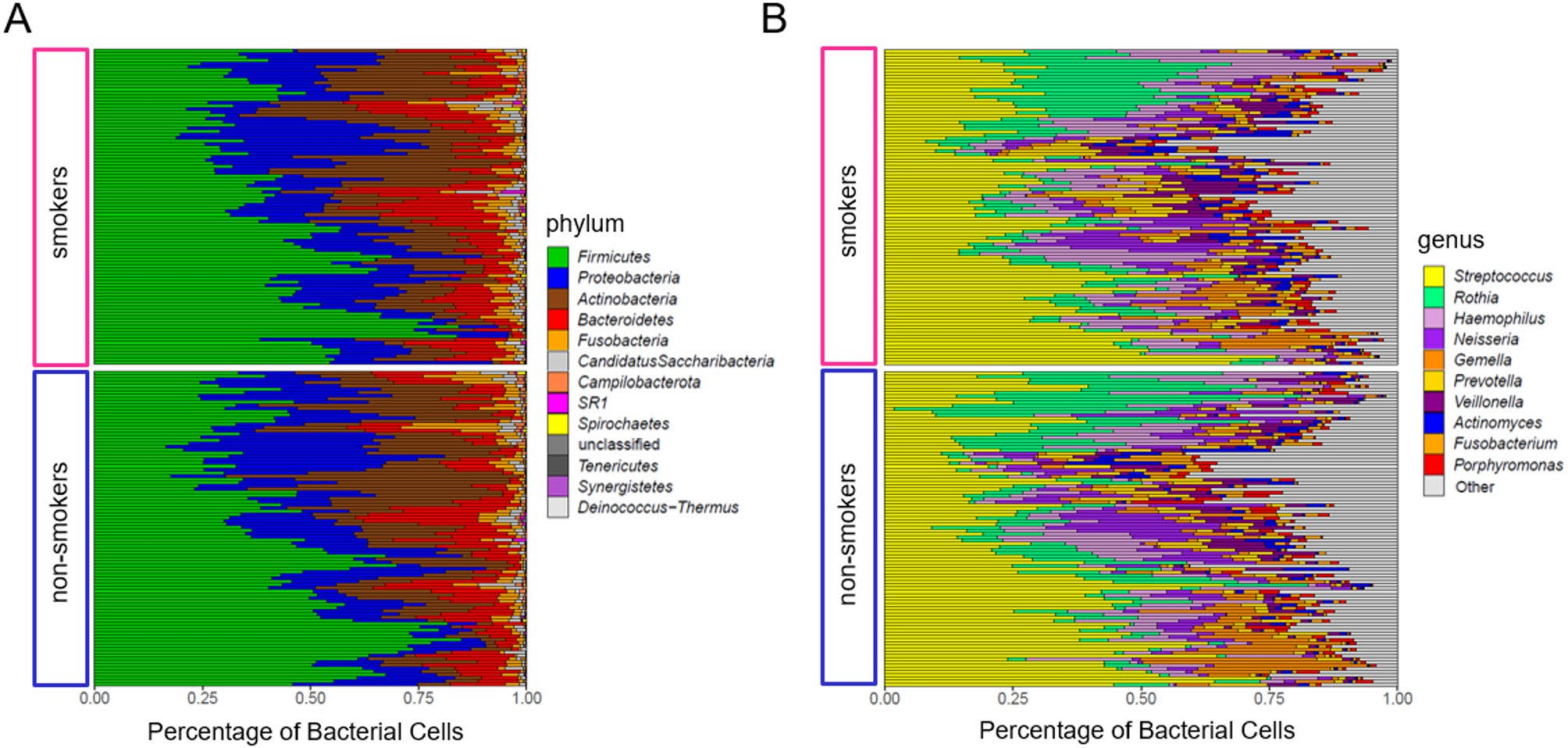
Taxonomic barplots on phylum- (**A**) and genus-level (**B**). (**A**) Relative abundances of bacterial cells for all phyla and (**B**) for the ten most abundant genera (calculated over all samples together) are depicted.

Overall, the buccal microbiome of the adolescents comprised 12 phyla, 78 families, 169 genera and 733 species-level taxa that could be identified. The most prominent bacterial phyla in the adolescents’ microbiomes were *Firmicutes*, *Proteobacteria*, *Actinobacteria*, *Bacteroidetes*, and *Fusobacteria*, together accounting for more than 99% of sequence reads on average (Fig. 3A). *Streptococcus* was on average the most abundant genus in smokers as well as in non-smokers (38 ± 15% in smokers, 39 ± 17% in non-smokers; Fig. 3B), with the *Streptococcus mitis/oralis* group as the most abundant species-level taxon (24 ± 15% in smokers, 26 ± 18% in non-smokers; Supplementary Fig. 6). The genera *Rothia (*8 ± 7% in smokers, 9 ± 9% in non-smokers), *Haemophilus*, *Neisseria*, and *Gemella* made up considerable proportions of the community in both smokers and non-smokers. None of these five most abundant genera differed significantly in abundance between smokers and non-smokers, as tested with DESeq2 (*p* adjusted > 0.05). The microbiota composition differed considerably between the individual participants (Fig. 3).

### Several taxa differed significantly between smokers and non-smokers

When testing for individual taxa that significantly differed between smokers and non-smokers, we identified two phyla, 12 families 15 genus-level taxa and 34 species-level taxa that were either more or less abundant in smokers, including higher levels of *Veillonella* (e.g. *V. atypica*), multiple *Actinomyces* species, *Dialister invisus*, *Atopobium parvulum*, *Streptococcus mutans* and *Prevotella melaninogenica* in the smoking group (Table 2). No taxon was found to be significantly associated with the number of bacterial cells in the sample.

**Table 2:**
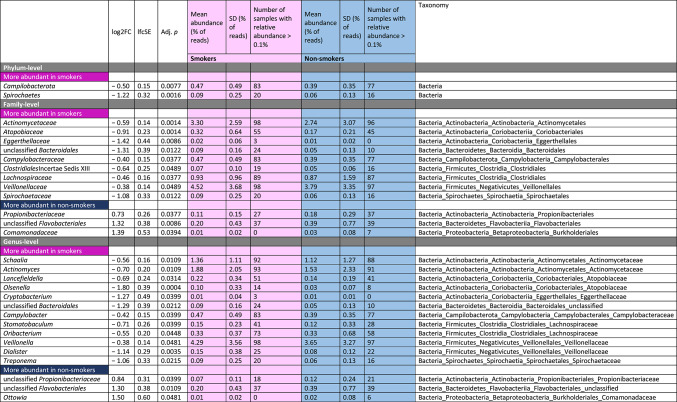

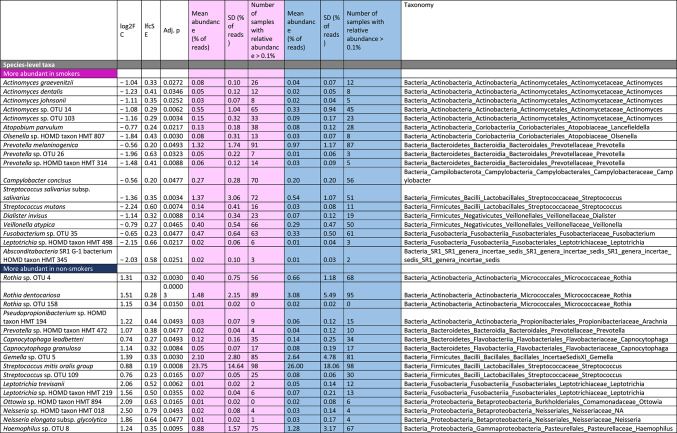
Results of DESeq2 analyses testing for significant differences at the levels of phylum, family, genus and species ordered by taxonomy.

## Discussion

To our knowledge, this is the first study to investigate the effects of smoking on the oral microbiome during the early stages of uptake—typically occurring in adolescence—rather than in adults with long-term established smoking habits^32^. In a recent German study, 15% of 14- to 20-year-olds already defined themselves as smokers^33,34^. While adult studies often include smokers that use more than 20 cigarettes per day^12,41^, in our study all of the adolescent smokers indicated lower smoking frequencies. In the present study, no effects of smoking frequency on the smokers’ oral microbiome could be demonstrated, likely due to the small sample sizes in some frequency groups. Projects to specifically examine those dose-dependent effects will likely need to be specifically targeted towards more equal representation of all smoking frequencies groups. As in our cohrt of pupils the exact duration since the first cigarette is necessarily confounded with age effects, we could not directly include this factor into our analysis, and no direct information on the duration of the participants’ smoking history was collected. Instead, we consider all smoking adolescents to be in the beginning stage of their smoking history. Combining all smokers, conventional cigarette, e-cigarette and “both types of cigarette”-smoker, into one group does of course preclude distinguishing the effects of e-cigarettes from that of tobacco smoke. However, most smokers in our study group stated that they used both conventional and e-cigarettes, hindering meaningful separation of the effects, especially when considering the limited subgroup sizes. The young age of the participants, as well as the overall low intensity of smoking, allow for an analysis of the earliest smoking-related effects on the composition of the oral microbiome. As all participants reported good oral hygiene practices and the absence of oral health issues, the observed changes predate smoking-related oral diseases. Oral health status was based solely on self-reported questionnaire data from the children, which may include inaccuracies. However, while potential undiagnosed conditions cannot be excluded, the large sample size should help to offset individual misreporting. The careful balancing of the dataset for various physiological characteristics of the participants enables reliable detection of these beginning microbiome changes at the onset of smoking. Besides other characteristics in our cohort, hormonal contraceptive use was recorded and carefully balanced between groups. Ongoing work in our group aims to elucidate its impact on the adolescent oral microbiome in greater detail. In contrast to most other studies on smoking-related changes in the oral microbiome, which are based on partial 16 S sequences, our analysis are based on full-length 16 S sequences, which allows for a higher taxonomic resolution and for classification at the species level for most sequences.

Independent of smoking, oral microbiome compositions of healthy adolescents have only rarely been analysed^42^. The oral communities of the adolescents in our study group are dominated by *Firmicutes*, mostly of the genus *Streptococcus*. But also *Proteobacteria*, in particular *Haemophilus* sp. and *Neisseria* sp., *Actinobacteria (*mostly *Rothia* sp.*)*, and *Bacteroidetes (Prevotella* sp.*)* made up large proportions of the microbiome. This resembles the composition of healthy oral microbiomes in adults^41,43,44^ and young adults^45^, and is also similar to the saliva microbiome of children^45^. The numbers of species-level taxa identified in the individual oral microbiomes of the adolescents in our study match the numbers that have been reported in young adolescents and adults^5,45,46^, indicating that the adolescent microbiome of our cohort of 14- to 20-year-olds resembles the adult microbiome in bacterial diversity and composition, which is in line with previous findings. The overall differences in oral microbiota composition of our adolescent study group are dominated by the differences between individuals, as is usually the case in the adult oral microbiome^5,47^. This matches existing studies showing that during the development of the oral microbiome from newborns to adults the bacterial diversity increases, reaching approximately adult levels during adolescence^45,48–50^. While host genetics has a discernible impact on the oral microbiome in young children, this influence decreases with age^51^ and is overlaid by an increasing effect of environmental factors, leading to a more and more individual-specific oral microbiome until adulthood^46,48,52^. Smoking is known as one of these factors, which have a significant impact on the oral microbiome, altering both the composition and diversity of bacterial communities. But its effect on the adolescent microbiome has been unknown so far.

Our study demonstrates that smoking exerts a discernible impact on the overall microbiome composition of adolescents which also occurs in adults^12,53^. If the observed effects are nicotine-dosage dependent as observed in airway samples of adults^54^, could not sufficiently be addressed here because of low participant numbers in sub-groups with different smoking frequencies. One of these changes detected in the present study is a small but significant increase in species-level taxon diversity in adolescent smokers. Previous studies in adult long-term smokers could not draw a clear picture regarding the alpha-diversity in the oral microbiome. While some studies observed a reduced diversity in the buccal microbiome of smokers^41^, others indicated the opposite effect^55^, or no difference in the buccal mucosa alpha diversity^14^. While some of this variation might be due to technical differences leading to differences in taxonomic resolution, or to differences in the numbers of participants, an influence of demographic differences, such as sex ratios, in the selected participant groups seems likely.

Several taxa exhibited significant increased relative abundance in smokers compared to non-smokers in our study group, including the genus *Veillonella* and *Veillonella atypica*,* Actinomyces* and several *Actinomyces* species, *Dialister invisus*,* Atopobium parvulum*,* Streptococcus mutans* and *Prevotella melaninogenica.* Most of these taxa have consistently been found to be more abundant in adult smokers as well, although often on a higher taxonomic level^56^. Interestingly, these taxa can mostly be classified as oral pathogens, which is in line with the current literature on the oral microbiome in adult smokers, as pathogens have already been shown to be more prevalent in smokers^13,57^.

As one example, *Veillonella* sp. have been reported to play a major role as a bridging organism in the development of commensal oral biofilms but were also suggested to promote disease progression by being the physical anchor and generator of favorable growth conditions for pathogens such as *P. gingivalis*, and in this way may act as “accessory pathogen”^58–61^. In addition, *Veillonella atypica* was also reported in a case study of a retropharyngeal abscess^62^. *Dialister invisus* was shown to be significantly associated with periodontal infections^63^, *Streptococcus mutans* is involved in the development of dental caries^64^, and *Atopobium parvulum*, was linked to dental caries^65^ and halitosis^66^, a condition characterised by bad smelling breath caused by a dysbiosis of the oral microbiome^67^. In addition to those taxa connected to oral infectious diseases and caries, the species *Prevotella melaninogenica* has been shown to be more common in saliva samples from patients with oral squamous cell carcinoma (OSCC) than in patients without OSCC^68^. Though, *Streptococcus mitis*, which likewise appeared with higher levels in patients with OSCC^68^, was identified more frequently in non-smokers in our analysis.

Several taxa appeared with a higher relative abundance in adolescent non-smokers, some of those having been observed in other studies associated with the oral microbiome of adult non-smokers as well. This refers to the taxa *Leptotrichia trevisanii* or the genus *Leptotrichia*^12^, *Neisseria* sp^13^. , *Haemophilus* sp^69^. , *Capnocytophaga* sp^12,70^. and *Gemella* sp^71^. Several species of the genus *Rothia* including *Rothia dentocariosa* were increased in the adolescent non-smokers in our cohort, while other studies in adults did not observe an association with non-smokers but rather an increase in the oral microbiome of smokers^14,71,72^. Interestingly, almost all of the taxa, which were more abundant in non-smoking adolescents in our study, namely the *Streptococcus mitis/oralis* group, *Neisseria* sp., *Haemophilus* sp., *Rothia dentocariosa*, and *Leptotrichia* sp. have been associated with oral health states elsewhere^63,73,74^. In summary, not only did our study detect similar smoking related-shifts in several bacterial taxa in adolescent microbiome as have been observed in adult smokers, but we also identified first smoking-associated changes towards the more pathogenic bacterial community in adolescents that have regularly been observed in adults.

Several mechanisms for microbial community shifts have been proposed in relation to smoking. Previous studies suggested, that smoking reduces oxygen, pH and immune cell-based defence^23,24,28,29^ and thereby favours growth of anaerobic, acid-tolerant, and pathogenic over commensal bacteria in the oral microbiomes of adult smokers^12,15,22,31^. In line with this, several obligate anaerobic bacterial taxa have been found with differential abundance in this study, (almost) all of which were significantly increased in smokers, indicating that reduced local oxygen tension, and the associated lower availability of oxygen for microbial metabolism, could play a role in oral biofilm formation of adolescent smokers as well. Several of these taxa have pathogenic potential and could shift the biofilm towards a more pathogenic state, especially since immune defence is reduced in smokers. These smoking-associated shifts in the oral microbiome could in the long term contribute to the development and progression of oral infectious diseases as it has been described for periodontitis and peri-implantitis in adults^17,19,57,75^. The oral microbiome of adolescents might already set the course for future health-associated microbiomes, such as shown for an association between the oral microbiome and weight gain^76^, celiac disease^77^ and Henoch-Schönlein purpura disease^78^. In summary, the smoking-associated oral microbiome changes captured in this study represent a very early stage of the adverse developments in the oral microbiome of smokers.

## Conclusion

The present study demonstrates the early onset of smoking-related changes in the oral microbiome. These findings related to oral health provide an illustrative example of the direct adverse health consequences of smoking that might seem more tangible to adolescents than the more drastic, severe consequences that can occur later in life. Highlighting these changes in the oral microbiome might be beneficial in raising awareness of the importance of smoking prevention.

## Supplementary Information

Below is the link to the electronic supplementary material.

Supplementary Material 1

## Data Availability

The sequence datasets generated and analysed during the current study have been deposited at NCBI (Sequence Read Archive) and are available under the BioProject PRJNA1140369.
