# Supplementary material for: Effects of cigarette smoking on the oral microbiome in adolescents
Source: Sci Rep. 2026 Jan 10;16:1348. doi: 10.1038/s41598-025-32650-2 (PMC12796446; doi:10.1038/s41598-025-32650-2)
Supplement: Supplementary file 1 — Supplementary Material 1 [file 41598_2025_32650_MOESM1_ESM.docx]

**Supplementary Table 1 (three pages)**

**Questionnaire**

Dear student, thank you very much for participating. Completing the questionnaire is, of course, voluntary. However, we kindly ask you to answer the following questions as completely as possible. All of your information will, of course, be treated confidentially. If you do not understand something, your parents can certainly help you. You are also welcome to ask us in person when you come in for your examination.

**Date: ______________**

**Age: _______ years**

**Gender: ________**

**Height: ________ cm**

**Weight: ________ kg**

1. **Symptoms of illness and SARS-CoV-2**

Do you currently have or have you had any of the following symptoms in the last 30 days:

Runny nose? □ **Yes □ No**

Sore throat? **□ Yes □ No**

Cough? **□ Yes □ No**

Shortness of breath when resting? **□ Yes □ No**

Shortness of breath during physical exertion? **□ Yes □ No**

Fever? **□ Yes □ No**

Fatigue? **□ Yes □ No**

Aches and pains? **□ Yes □ No**

Diarrhea? **□ Yes □ No**

Skin rash? **□ Yes □ No**

Loss of taste or smell? **□ Yes □ No**

*(Have you had difficulty tasting or smelling?)*

**Have you ever had a throat or nose swab test for SARS-CoV-2 (‘coronavirus test’)?**

□ **Yes** □ **No**

If yes, when? ____________________ Was the coronavirus detectable? **□ Yes □ No**

**Have you ever had a blood test for SARS-CoV-2 (‘coronavirus antibody test’)?**

**□ Yes □ No**

If yes, when? ____________________

Were antibodies against SARS-CoV-2 detectable?

**□ Yes □ No**

1. **Pre-existing conditions: Do you have a medical condition that requires you to visit your doctor regularly?**

**□ Yes □ No**

If yes, what is the name of the condition?

**Do you take medication regularly (including the pill)?** **□ Yes □ No**

If yes, how often? □ Every day

□ Several times a week

□ Once a week

□ Less than once a week

What are the names of the medications?

**____________________________________________________________________________**

1. **Oral hygiene and smoking habits**

**How often do you brush your teeth?**

□ Twice a day or more

□ Once a day

□ Several times a week

□ Once a week

**Do you use fluoride toothpaste?**

□ Toothpaste with fluoride

□ Toothpaste without fluoride

□ No toothpaste

**Do your gums bleed when you brush your teeth?**

□ Never

□ Occasionally

□ Always

**Have you ever been, or are you currently being treated by a dentist for gum disease?**

□ Yes

□ No

Next, we would like to ask you about your smoking habits. Please answer honestly. Your answers will be treated confidentially!

**Have you ever smoked/vaped/vaporised a cigarette or e-cigarette?**

□ Cigarette □ E-cigarette □ Never

**If yes, how often on average have you smoked/vaped/vaporised a cigarette or e-cigarette in the last 3 months?**

**Cigarette**

□ Never

□ Less than once a month

□ Once a month

□ Several times a month

□ Several times a week

□ Every day

□ More than 20 cigarettes a day

**E-cigarette**

□ Never

□ Less than once a month

□ Once a month

□ Several times a month

□ Several times a week

□ Every day

□ More than 20 cigarettes a day

**Supplementary Table 2 (two pages)**

Sixty-one contaminating species or OTUs that were removed from the data set due to high abundance in negative controls. During generation of the phylogenetic tree, seven additional species-level taxa were removed due to short or inaccurate alignment to the 16S rDNA sequences.

| **domain** | **phylum** | **class** | **order** | **family** | **genus** | **Species-level taxon** |
| --- | --- | --- | --- | --- | --- | --- |
| *Contaminants that were removed from the data set due to high abundance in negative controls* | | | | | | |
| Bacteria | Actinobacteria | Actinobacteria | Bifidobacteriales | Bifidobacteriaceae | Bifidobacterium | Bifidobacterium_pseudocatenulatum |
| Bacteria | Actinobacteria | Actinobacteria | Bifidobacteriales | Bifidobacteriaceae | Gardnerella | Gardnerella_vaginalis |
| Bacteria | Actinobacteria | Actinobacteria | Mycobacteriales | Corynebacteriaceae | Corynebacterium | Corynebacterium_coyleae |
| Bacteria | Actinobacteria | Actinobacteria | Mycobacteriales | Corynebacteriaceae | Corynebacterium | Corynebacterium_mucifaciens |
| Bacteria | Actinobacteria | Actinobacteria | Mycobacteriales | Nocardiaceae | Rhodococcus | OTU_50 |
| Bacteria | Actinobacteria | Actinobacteria | Propionibacteriales | Propionibacteriaceae | Cutibacterium | OTU_19 |
| Bacteria | Bacteroidetes | Bacteroidia | Bacteroidales | NA | NA | Bacteroidetes_[G-3]_bacterium_HOMD_taxon_HMT_365 |
| Bacteria | Bacteroidetes | Bacteroidia | Bacteroidales | Porphyromonadaceae | Parabacteroides | Parabacteroides_goldsteinii |
| Bacteria | Bacteroidetes | Bacteroidia | Bacteroidales | Prevotellaceae | Prevotella | Prevotella_baroniae |
| Bacteria | Bacteroidetes | Bacteroidia | Bacteroidales | Prevotellaceae | Prevotella | Prevotella_corporis |
| Bacteria | Bacteroidetes | Bacteroidia | Bacteroidales | Prevotellaceae | Prevotella | Prevotella_multiformis |
| Bacteria | Bacteroidetes | Bacteroidia | Bacteroidales | Prevotellaceae | Prevotella | Prevotella_sp._HOMD_taxon_HMT_443 |
| Bacteria | Bacteroidetes | Flavobacteriia | Flavobacteriales | Flavobacteriaceae | Flavobacterium | Flavobacterium_hercynium |
| Bacteria | Deinococcus-Thermus | Deinococci | Trueperales | Trueperaceae | Truepera | Truepera_radiovictrix |
| Bacteria | Firmicutes | Bacilli | Bacillales | Bacillaceae1 | NA | Anaerobacillus_alkalidiazotrophicus |
| Bacteria | Firmicutes | Bacilli | Bacillales | Paenibacillaceae1 | Paenibacillus | Paenibacillus_barengoltzii |
| Bacteria | Firmicutes | Bacilli | Bacillales | Paenibacillaceae1 | Paenibacillus | Paenibacillus_phoenicis |
| Bacteria | Firmicutes | Bacilli | Bacillales | Staphylococcaceae | Staphylococcus | OTU_113 |
| Bacteria | Firmicutes | Bacilli | Bacillales | Staphylococcaceae | Staphylococcus | Staphylococcus_aureus |
| Bacteria | Firmicutes | Bacilli | Bacillales | Staphylococcaceae | Staphylococcus | Staphylococcus_epidermidis |
| Bacteria | Firmicutes | Bacilli | Lactobacillales | Lactobacillaceae | Ligilactobacillus | OTU_66 |
| Bacteria | Firmicutes | Bacilli | Lactobacillales | Streptococcaceae | Streptococcus | Streptococcus_equi |
| Bacteria | Firmicutes | Clostridia | Clostridiales | Lachnospiraceae | Butyrivibrio | Butyrivibrio_sp._HOMD_taxon_HMT_455 |
| Bacteria | Firmicutes | Clostridia | Clostridiales | Lachnospiraceae | NA | Butyrivibrio_sp._HOMD_taxon_HMT_080 |
| Bacteria | Firmicutes | Clostridia | Clostridiales | Peptoniphilaceae | Anaerococcus | Anaerococcus_lactolyticus |
| Bacteria | Firmicutes | Clostridia | Clostridiales | Peptoniphilaceae | Anaerococcus | Anaerococcus_murdochii |
| Bacteria | Firmicutes | Clostridia | Clostridiales | Peptoniphilaceae | Anaerococcus | Anaerococcus_prevotii |
| Bacteria | Firmicutes | Clostridia | Clostridiales | Peptoniphilaceae | Finegoldia | Finegoldia_magna |
| Bacteria | Firmicutes | Clostridia | Clostridiales | Peptoniphilaceae | Peptoniphilus | OTU_589 |
| Bacteria | Firmicutes | Clostridia | Clostridiales | Peptoniphilaceae | Peptoniphilus | Peptoniphilus_sp._HOMD_taxon_HMT_375 |
| Bacteria | Firmicutes | Erysipelotrichia | Erysipelotrichales | Erysipelotrichaceae | Erysipelothrix | OTU_217 |
| Bacteria | Firmicutes | Negativicutes | Selenomonadales | Selenomonadaceae | Selenomonas | OTU_258 |
| Bacteria | Firmicutes | Negativicutes | Selenomonadales | Selenomonadaceae | Selenomonas | Selenomonas_sp._HOMD_taxon_HMT_134 |
| Bacteria | Firmicutes | Negativicutes | Selenomonadales | Selenomonadaceae | Selenomonas | Selenomonas_sp._HOMD_taxon_HMT_138 |
| Bacteria | Proteobacteria | Alphaproteobacteria | Rhodospirillales | Azospirillaceae | Nitrospirillum | OTU_136 |
| Bacteria | Proteobacteria | Betaproteobacteria | Burkholderiales | Burkholderiaceae | Cupriavidus | Cupriavidus_basilensis |
| Bacteria | Proteobacteria | Betaproteobacteria | Burkholderiales | Burkholderiaceae | Paraburkholderia | OTU_165 |
| Bacteria | Proteobacteria | Betaproteobacteria | Burkholderiales | Burkholderiaceae | Paraburkholderia | Paraburkholderia_bryophila |
| Bacteria | Proteobacteria | Betaproteobacteria | Burkholderiales | Burkholderiaceae | Paraburkholderia | Paraburkholderia_insulsa |
| Bacteria | Proteobacteria | Betaproteobacteria | Burkholderiales | Burkholderiaceae | Ralstonia | OTU_106 |
| Bacteria | Proteobacteria | Betaproteobacteria | Burkholderiales | Burkholderiaceae | Ralstonia | Ralstonia_mannitolilytica |
| Bacteria | Proteobacteria | Betaproteobacteria | Burkholderiales | Burkholderiaceae | Ralstonia | Ralstonia_pickettii |
| Bacteria | Proteobacteria | Betaproteobacteria | Burkholderiales | Comamonadaceae | Pelomonas | Pelomonas_puraquae |
| Bacteria | Proteobacteria | Betaproteobacteria | Neisseriales | Neisseriaceae | Eikenella | OTU_529 |
| Bacteria | Proteobacteria | Betaproteobacteria | Neisseriales | Neisseriaceae | NA | Neisseriaceae_[G-1]_bacterium_HOMD_taxon_HMT_327 |
| Bacteria | Proteobacteria | Betaproteobacteria | Rhodocyclales | Rhodocyclaceae | Oryzomicrobium | Rhodocyclus_sp._HOMD_taxon_HMT_028 |
| Bacteria | Proteobacteria | Gammaproteobacteria | Enterobacterales | Enterobacteriaceae | Enterobacter | OTU_34 |
| Bacteria | Proteobacteria | Gammaproteobacteria | Oceanospirillales | Halomonadaceae | Halomonas | Halomonas_desiderata |
| Bacteria | Proteobacteria | Gammaproteobacteria | Oceanospirillales | Halomonadaceae | Halomonas | Halomonas_johnsoniae |
| Bacteria | Proteobacteria | Gammaproteobacteria | Oceanospirillales | Halomonadaceae | Halomonas | Halomonas_stevensii |
| Bacteria | Proteobacteria | Gammaproteobacteria | Pseudomonadales | Moraxellaceae | Acinetobacter | OTU_390 |
| Bacteria | Proteobacteria | Gammaproteobacteria | Pseudomonadales | Moraxellaceae | Enhydrobacter | Enhydrobacter_aerosaccus |
| Bacteria | Proteobacteria | Gammaproteobacteria | Pseudomonadales | Pseudomonadaceae | Pseudomonas | OTU_21 |
| Bacteria | Proteobacteria | Gammaproteobacteria | Pseudomonadales | Pseudomonadaceae | Pseudomonas | Pseudomonas_poae |
| Bacteria | Proteobacteria | Gammaproteobacteria | Pseudomonadales | Pseudomonadaceae | Pseudomonas | Pseudomonas_pseudoalcaligenes |
| Bacteria | Proteobacteria | Gammaproteobacteria | Pseudomonadales | Pseudomonadaceae | Pseudomonas | Pseudomonas_psychrophila |
| Bacteria | Proteobacteria | Gammaproteobacteria | Pseudomonadales | Pseudomonadaceae | Pseudomonas | Pseudomonas_yamanorum |
| Bacteria | Proteobacteria | Gammaproteobacteria | Vibrionales | Vibrionaceae | Photobacterium | OTU_388 |
| Bacteria | Proteobacteria | Gammaproteobacteria | Xanthomonadales | Xanthomonadaceae | Stenotrophomonas | Stenotrophomonas_maltophilia |
| Bacteria | Spirochaetes | Spirochaetia | Spirochaetales | Spirochaetaceae | Treponema | Treponema_socranskii_subsp._socranskii |
| Bacteria | Spirochaetes | Spirochaetia | Spirochaetales | Spirochaetaceae | Treponema | Treponema_sp._HOMD_taxon_HMT_951 |
|  |  |  |  |  |  |  |
| *Contaminants that were removed from the data set due to low quality alignment to the16S rDNA sequences)* | | | | | | |
| Bacteria | Firmicutes | Clostridia | Clostridiales | Lachnospiraceae | Lachnoanaerobaculum | Lachnoanaerobaculum_orale |
| Bacteria | Proteobacteria | Betaproteobacteria | Burkholderiales | Burkholderiaceae | Paraburkholderia | Paraburkholderia_fungorum |
| Bacteria | Proteobacteria | Betaproteobacteria | Neisseriales | Neisseriaceae | Neisseria | OTU_36 |
| Bacteria | Proteobacteria | Gammaproteobacteria | Gammaproteobacteria_incertae_sedis | CandidatusCarsonella | CandidatusCarsonella | OTU_397 |
| Bacteria | Proteobacteria | Gammaproteobacteria | Gammaproteobacteria_incertae_sedis | CandidatusCarsonella | CandidatusCarsonella | OTU_433 |
| Bacteria | Proteobacteria | Gammaproteobacteria | Gammaproteobacteria_incertae_sedis | CandidatusCarsonella | CandidatusCarsonella | OTU_72 |
| Bacteria | Proteobacteria | Gammaproteobacteria | NA | NA | NA | OTU_242 |

**Supplementary Methods**

*DNA extraction*

DNA was extracted with a customized protocol under DNA-free conditions in an air-clean workbench (AirClean 600 PCR workstation, Starlab, Hamburg, Germany). The whole sample material, including storage buffer and cotton swab, was transferred to Lysing Matrix E 2 ml tubes (MP Biomedicals, Eschwege, Germany). Cells were ruptured in three cycles of 6500 rpm for 30 s in a Precellys 24 homogenizer (Bertin technologies, Frankfurt am Main, Germany) and cooled on ice for 5 min in between rupturing steps. The beads were sedimented by centrifugation (5 min, 14000 x g). The supernatant (ca. 500 µl) was mixed with an equal volume of ethanol and applied to QIAamp spin columns (Qiagen, Hilden, Germany). Subsequent DNA isolation steps were based on the QIAamp DNA Blood Mini Kit (Qiagen) following protocol “DNA Purification from Blood or Body Fluids” starting from application to the spin column. As the initial volume exceeded the capacity of the column, the samples were added in two steps of 500 µl each, followed by centrifugation at 6000 x g for 1 min. To reduce the risk of contamination, a new collection tube was used after each of the kit-specific wash steps. The isolated DNA was eluted with 50 µl of PCR-grade water. To increase the final DNA concentration, the eluate was applied to the column again for a second elution step. The isolated DNA was stored at -20°C until further use.

*Full-length 16S rDNA amplicon generation and sequencing*

In preparation of full-length 16S rRNA gene sequencing using PacBio SMRT technology, V1-V9 regions of bacterial 16S rRNA genes were amplified from the isolated DNA by polymerase chain reaction with the PacBio-recommended, bacteria-specific primers 27F (AGRGTTYGATYMTGGCTCAG) and 1492R (RGYTACCTTGTTACGACTT). Reaction mixtures contained 25 µl KAPA HiFi HotStart ReadyMix (Roche, Grenzach-Wyhlen, Germany) per 50 µl of reaction. PCR was performed with an initial denaturation step of 3 min at 95°C, followed by 23 cycles of 30 sec at 95°C, 30 sec at 55°C and 90 sec at 72°C, and a terminal elongation for 10 min at 72°C.

Amplicons were purified using AMPure PB beads (PacBio, Menlo Park, CA, USA) according to the PacBio-recommended protocol and final DNA amounts per sample were measured with the Qubit dsDNA HS Assay Kit (Thermo Fisher Scientific, Waltham, MA, USA). Subsequent preparation for sequencing, including barcode ligation, followed the PacBio protocol “Procedure & Checklist - Preparing SMRTbell® Libraries using PacBio Barcoded Overhang Adapters for Multiplexing Amplicons” (version 06, Jan 2021) using chemicals of the SMRTbell® Express Template Prep Kit 2.0. Whenever possible, 50 ng of amplicon DNA per sample were processed in the library preparation. In case of samples with lower DNA concentrations, equal amounts of DNA for all samples on one SMRT cell were processed. Sixteen samples were prepared to be sequenced on one SMRT cell. Sequencing was performed with the Sequel Binding and Sequencing kits 3.0 according to PacBio-recommendation for full-length 16S sequencing.

*Sequence analyses*

Circular consensus sequences (CCS) with a minimum predicted accuracy of 0.999 were computed using SMRT Link v10.1 (PacBio). Further analysis was based on an in-house pipeline (Desch et al 2020). Sequences were demultiplexed and barcodes as well as primer sequences were removed with Tagcleaner 0.16. Sequences that had more than one mismatch to one or both of the barcode sequences or more than five mismatches to one or both of the primer sequences were rejected. The sequences were dereplicated using vsearch 2.21.1 (Rognes et al 2016). We used BLAST on a modified bacteria-only version of the SILVA SSU Ref_NR 99 database version 132 (Quast et al 2013), enriched with human Oral Microbiome Database eHOMD 16S rRNA RefSeq Version 15.1 sequences (Desch et al 2020), as well as version LTPs132_SSU of the All-Species Living Tree Project (LTP) database, supplemented with HOMD unnamed and phylotype sequences, to identify sequences to species level. Sequences for which identification based on each of these two databases was unambiguous, and for which both classification attempts led to the same species designation, were identified to species. The remaining sequences were divided into taxonomic operational units (OTUs), which had a minimum intra-OTU comparison of 97 using UPARSE as implemented in USEARCH 10.0.240 (Edgar 2013). To obtain taxonomic classification above the species level, representative sequences of OTUs and all sequences classified to species were also classified with RDP classifier 2.12 (Wang et al 2007) at a minimum bootstrap support value of 0.8. The representative sequences of these OTUs were tested to exclude contamination with mitochondrial and chloroplastic sequences. Sequences not classified at the class level or below were only retained if the SILVA database contained at least 50 sequences with a length of at least 95% identity over a minimum length of 399 bp. As a basis for all statistical analyses, the list of all remaining OTUs was merged with the list of identified species to form a final set of species-level taxa. Statistical analyses of the microbiota composition were based on the counts taxon-specific 16S sequences in the individual samples. The number of bacterial cells of each species-level taxon were extrapolated based on the 16S copy numbers from rrnDB version 5.6 (Stoddard et al 2015).

Based on the clean cotton swab control samples, 61 species-level taxa were removed as likely contaminants prior to all further analyses (Supplementary Table 2). Species-level taxa were defined as contaminants based on following criteria:

1. Species-level taxa with a maximal read number in the empty samples exceeding one-tenth of the maximal read number in samples were removed.
2. A subsequent filtering step removed species-level taxa which only had an outlier maximum in one sample and otherwise met criterion 1.
3. Species-level taxa with similar mean read counts in samples and controls were removed (mean less than five times larger in samples compared to empty samples).
4. Furthermore, seven species-level taxa were removed due to short or inaccurate alignment to 16S rDNA sequences (Supplementary Table 2).

*Determination of sample-specific extraction bias*

The sample-specific extraction efficiency index was calculated based on the spike-in species ratio. The ratio was calculated as the ratio of total *Allobacillus* sequence read numbers to total *Imtechella* sequence read numbers.

This extraction efficiency index differed slightly but significantly between smokers and non-smokers (independent t-test: p = 0.003, Cohen’s d = 0.43, Supplementary Figure 1 A). The extraction efficiency was significantly different between non-smokers and participants that smoke both conventional and e-cigarette (in a comparison between only-conventional, only-e-cigarettes, smokers of both and non-smokers; ANOVA: p = 0.0176, Tukey multiple comparisons of means on 95% family-wise confidence level: p = 0.02; Supplementary Figure 1 B). It was not significantly different between different smoking frequencies (ANOVA: p = 0.0624). The extraction efficiency index was included into the statistical models for PERMANOVA and DESeq2 analyses as representative for the potential bias in cell lysis.


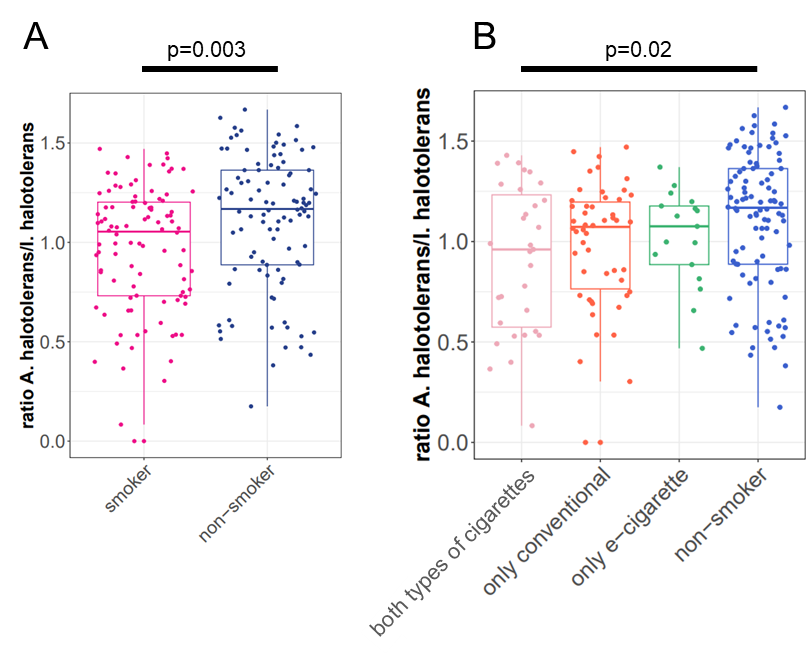

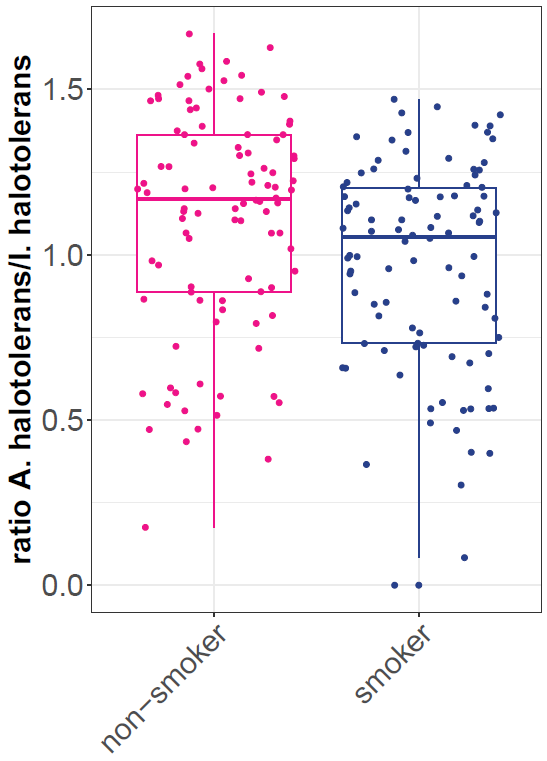

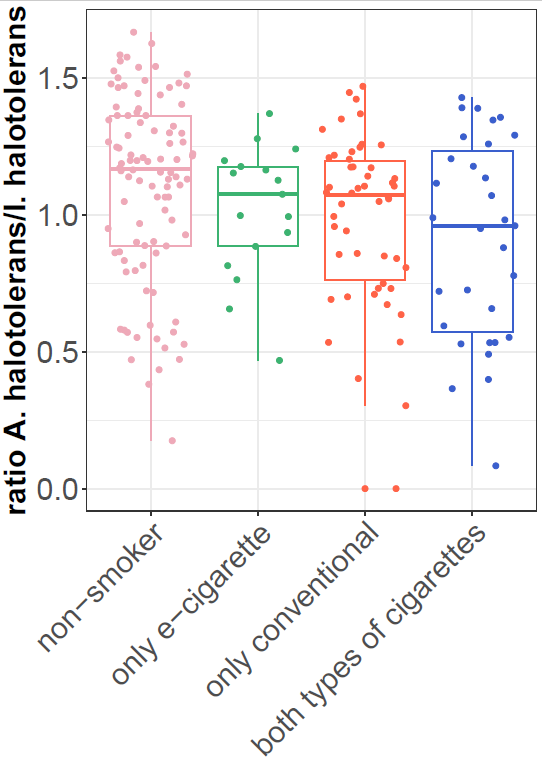


A

B

p=0.003

p=0.02

**Supplementary Figure 1: Box plots on the extraction efficiency index.**

Sample-specific extraction efficiency index, calculated as the ratio of absolute sequence reads from two spike-in species with different cell wall characteristics, shown as box plots. Boxes indicate the median (50th percentile), 25th and 75th percentiles, and whiskers reach to 1.5 times the interquartile ranges.

*Statistical calculations*

Descriptive statistics, tests for homogeneity of variance and normal distribution, independent t-test, Wilcoxon rank-sum test, ANOVA, Kruskal-Wallis rank sum test, and effect sizes (Cohen’s d) were calculated with the R packages car (version 3.1.1), stats (version 4.1.0) and lsr (version 0.5.2).

**Supplementary Results**


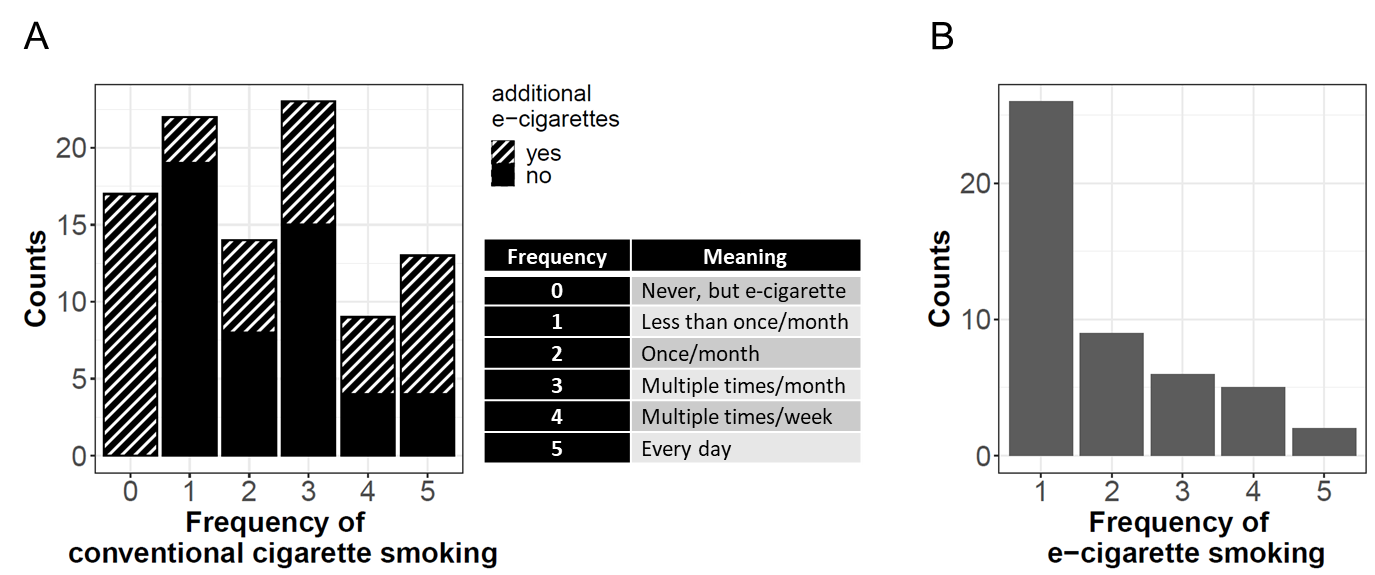


**Supplementary Figure 2: Counts of participants by smoking frequency of conventional cigarettes.** A) Eighty-one subjects smoked conventional cigarettes. Frequencies ranged from less than once per month (category 1, n = 22) to every day (category 5, n = 13). No participant smoked more than 20 cigarettes / day. B) Counts of e-cigarette using participants by e-cigarette smoking frequency. Frequency categories defined as in A. Most (35 out of 48) e-cigarette smokers belong to e-cigarette smoking frequency categories 1 and 2, hence use e-cigarettes not more than once a month.

**
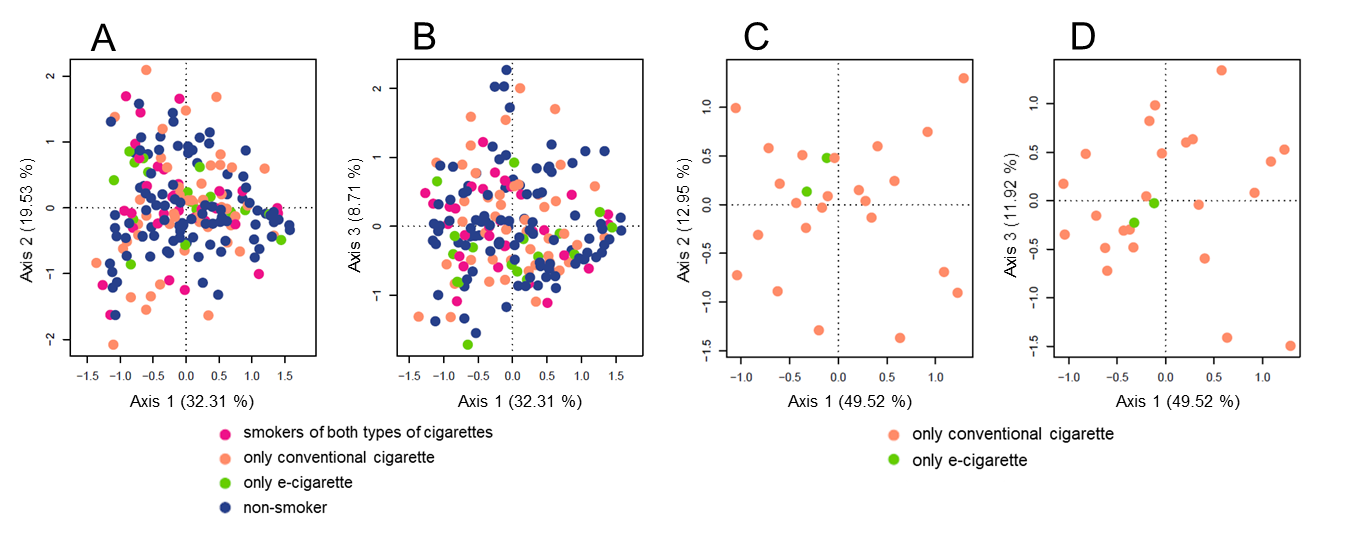
**

**Supplementary Figure 3: PCoA based on weighted UniFrac distances depicting clustering of bacterial communities according to smoking of conventional or e-cigarettes.** A, B). Comparison according to more detailed classifications of smoking behaviour (non-smoker, only conventional, only e-cigarette, both types of cigarettes) did not identify significant differences between the bacterial communities (PERMANOVA, p > 0.05). C, D) Likewise, comparison including only frequent smokers (categories 3, 4, and 5 corresponding to several times a month) of conventional cigarettes (orange, n = 23) and e-cigarettes (green, n = 2) (PERMANOVA, p > 0.05) did not identify significant differences.


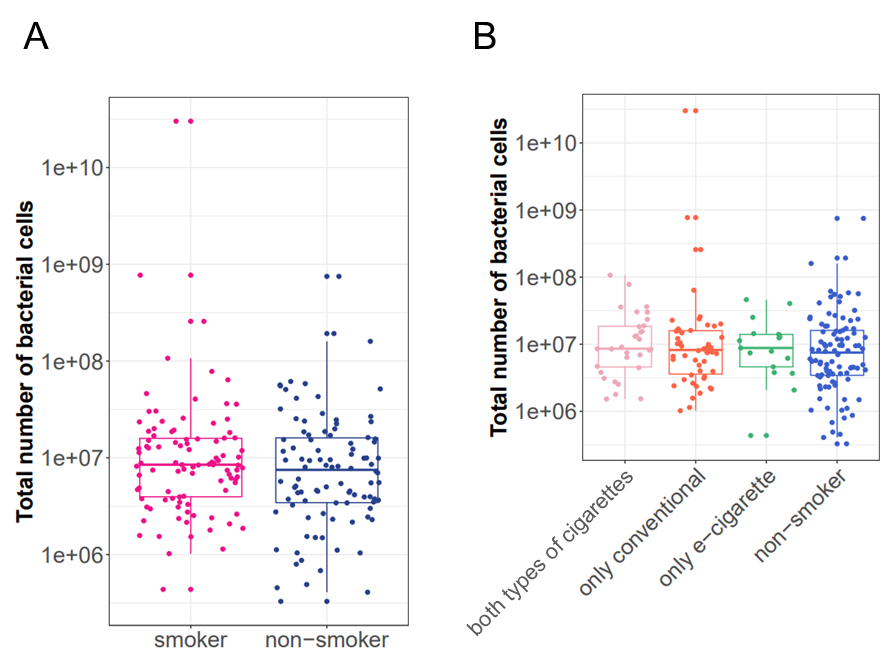


**Supplementary Figure 4: Total number of bacterial cells in buccal swabs from smokers and non-smokers.**

A) Buccal swaps contained similar numbers of bacterial cells in samples from smokers and non-smokers (t-test: p > 0.05). B) Likewise, bacterial cell numbers did not differ between different groups of smoking behaviour (ANOVA: p > 0.05). Numbers of bacterial cells were calculated from the defined number of *Imtechella* cells in the starting sample corrected for 16S copies per bacterial cell. Boxes indicate the median (50th percentile), 25th and 75th percentiles, and whiskers reach to 1.5 times the interquartile ranges.


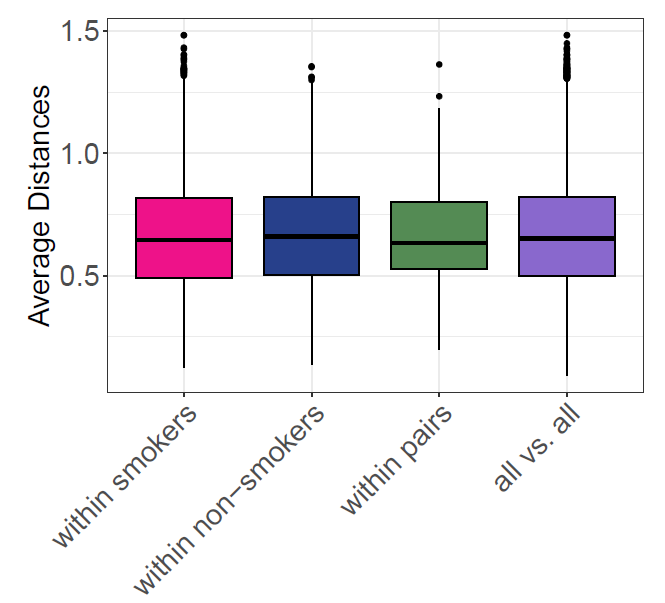


**Supplementary Figure 5: Weighted UniFrac distances between groups of participants.**

Boxplots depict the pairwise weighted UniFrac distances between all samples within the group of smokers, the group of non-smokers, within matched pairs, or within the complete group of participants. Boxes indicate the median (50th percentile), 25th and 75th percentiles, and whiskers reach to 1.5 times the interquartile ranges. Outliers are depicted as black dots. Distances did not significantly differ between the groups (ANOVA, p > 0.05).


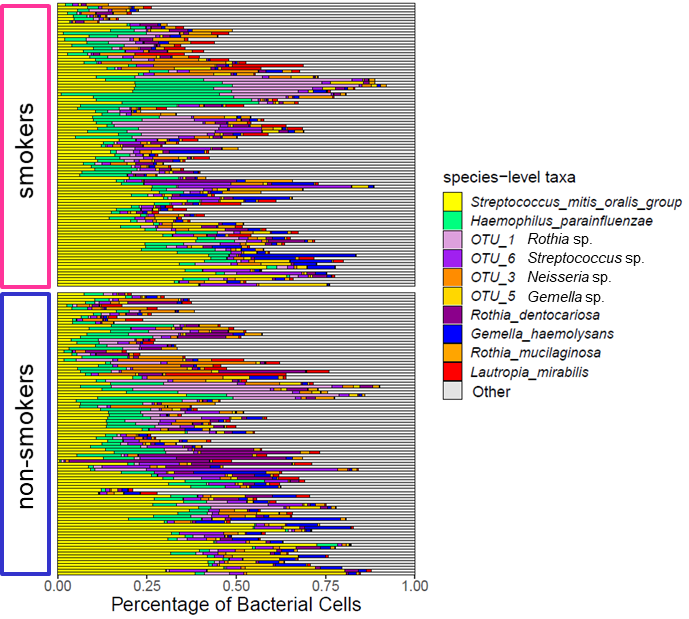


**Supplementary Figure 6: Most abundant species-level taxa.**

Taxonomic barplots on the species level depicting the relative abundances of bacterial cells for the ten most abundant species-level taxa as calculated over all samples.

**Supplementary References**

Desch A, Freifrau von Maltzahn N, Stumpp N, Dalton M, Yang I, Stiesch M (2020). Biofilm formation on zirconia and titanium over time-An in vivo model study. *Clin Oral Implants Res* **31:** 865-880.

Edgar RC (2013). UPARSE: highly accurate OTU sequences from microbial amplicon reads. *Nat Methods* **10:** 996-998.

Quast C, Pruesse E, Yilmaz P, Gerken J, Schweer T, Yarza P *et al* (2013). The SILVA ribosomal RNA gene database project: improved data processing and web-based tools. *Nucleic Acids Res* **41:** D590-596.

Rognes T, Flouri T, Nichols B, Quince C, Mahe F (2016). VSEARCH: a versatile open source tool for metagenomics. *PeerJ* **4:** e2584.

Stoddard SF, Smith BJ, Hein R, Roller BR, Schmidt TM (2015). rrnDB: improved tools for interpreting rRNA gene abundance in bacteria and archaea and a new foundation for future development. *Nucleic Acids Res* **43:** D593-598.

Wang Q, Garrity GM, Tiedje JM, Cole JR (2007). Naive Bayesian classifier for rapid assignment of rRNA sequences into the new bacterial taxonomy. *Appl Environ Microb* **73:** 5261-5267.
